# Characterization of Home Working Population during COVID-19 Emergency: A Cross-Sectional Analysis

**DOI:** 10.3390/ijerph17176284

**Published:** 2020-08-28

**Authors:** Antimo Moretti, Fabrizio Menna, Milena Aulicino, Marco Paoletta, Sara Liguori, Giovanni Iolascon

**Affiliations:** Department of Medical and Surgical Specialties and Dentistry, University of Campania “Luigi Vanvitelli”, 80138 Naples, Italy; fabrizio.menna@studenti.unicampania.it (F.M.); milena.aulicino@alice.it (M.A.); marco.paoletta@unicampania.it (M.P.); sara.liguori@unicampania.it (S.L.); giovanni.iolascon@unicampania.it (G.I.)

**Keywords:** home working, smart working, musculoskeletal pain, low back pain, neck pain, job satisfaction, occupational stress, workplace, work performance, COVID-19

## Abstract

Evidence about the characterization of home workers in terms of both work-related outcomes and health issues is lacking. The purpose of this cross-sectional study was to examine the impact of home working on perceived job productivity and satisfaction, work-related stress, and musculoskeletal (MSK) issues. We included 51 mobile workers, collecting data about demographic characteristics, working experience, job productivity, and stress. Job satisfaction was assessed through the Utrecht Work Engagement Scale (UWES), while MSK pain was investigated by the Brief Pain Inventory (BPI) and Fear Avoidance Beliefs Questionnaire (FABQ). Moreover, a home workplace analysis had to be carried out according to current Italian regulations. Participants declared that they were less productive (39.2%) but less stressed (39.2%) and equally satisfied (51%) compared to the time of office working. Regarding MSK disorders, low back pain (LBP) was referred by 41.2% of home workers and neck pain by 23.5% of them. Neck pain worsened in 50% of home workers, while LBP did not exacerbate in 47.6% of cases. Home workers with MSK pain reported a lower job satisfaction. Depending on our data, the home environment seems to be not adequate in the mobile worker population, with an increased risk for mental health and MSK problems, particularly affecting the spine. Addressing these issues can significantly reduce risks for health, thus, improving job productivity and satisfaction and reducing cost.

## 1. Introduction

The COVID-19 health emergency has profoundly changed working life. To minimize physical contact among individuals and prevent new infections, many companies implemented “mobile working” or “home working” or “remote working”, a form of carrying out a job without specific place of work restrictions, with the possible use of technological tools [1].

In 2017, Italy had the lowest percentage of remote workers across all Europe [1], and this percentage amounted to about 8% of total employment at the end of April 2020.

During the COVID-19 pandemic, the number of remote workers increased by 69% in Italy, while it has been estimated that about 81% of the worldwide workforce has been affected by workplace changes [2].

For most remote employees, it has probably been the first experience. Among advantages, there are reduced commuting time, possible productivity gains, increased staff motivation, better work–life balance, and better control over time schedule, while among disadvantages there are difficulties monitoring performance, cost of working from home, communication problems, no clear separation between home and work tasks, and unsuitability with all works [3,4].

The home environment is likely to be faulty in many aspects in comparison to the workplace. In particular, the absence of ergonomic office furniture at home may impede the adoption of a healthy posture and may promote the onset of musculoskeletal (MSK) disorders [5,6]. Working in a sedentary position for prolonged periods increases the risk of neck pain and/or low back pain (LBP) [7,8].

Home working may cause also stress, anxiety, and isolation, which influences job effectiveness, well-being, and work–life balance [9,10].

Even if the effects of home working on various aspects (e.g., quality of life, health and safety, and productivity) have been also investigated, this research area is still developing. Whilst the psychological benefits of home working—e.g., higher work engagement, work-related flow, and connectivity among staff—can attract many organizations to consider its implementation, the negative impacts such as blurred work–home boundary, fatigue, and mental demands should be addressed when/if home working is implemented [11]. For many workers, the opportunity to work from a home office makes everyday life easier. Among positive effects the most expected are higher efficiency at work, better concentration, reduction of psychological stress, and a better family life [12]. Working at home permits a better work–life balance, and this is important for workers caring for sick family members or children, but this results in little time for personal leisure activities [13,14]. On the other side, there are negative effects associated with remote working. For example, it has been found that home workers experience overlaps between work and home lives [15]. Moreover, they often experienced increased irritability and negative emotions, which were attributed to social isolation and being unable to share the troubles at work and find possible solutions with colleagues [16]. 

However, studies concerning the characterization of the mobile worker population in terms of both work-related outcomes and health issues are lacking.

The aim of this study is to investigate the role of home working on job satisfaction, occupational stress, perceived productivity, and MSK issues.

## 2. Materials and Methods 

### 2.1. Study Participants and Procedures

A population of mobile workers was included in the present cross-sectional study. Participants were contacted by phone; they received a full explanation of the study and signed an informed consent about privacy regulations regarding their personal data. All individuals were employed as administrative officers that moved to work remotely since the beginning of COVID-19 health emergency. Office work lasted for 8 h a day, with a 1 h lunch break. This study was conducted in accordance with the Declaration of Helsinki and its later amendments.

### 2.2. Measures

We prepared a questionnaire consisting of 12 items. We investigated employees’ subjective data such as age, gender, weight, height, education, job levels, and cohabitants, in particular children. Subsequently, we asked participants about their previous remote working experience, focusing on the kind of job and its differences from traditional work (tasks, schedule, and salary). We also included questions about productivity, work-related stress, and job satisfaction. In particular, we asked about factors that might improve productivity (saved travel time to go to the office, time flexibility, autonomy, reconciliation of work life with personal and family life, enhanced attention) or might decrease it (distractions in the domestic environment such as children to look after, planning difficulties, impaired interaction with colleagues, technical failures). For questions about advantages and disadvantages of working at home on job productivity, it was possible to choose multiple answers. Finally, the workers were asked whether they would continue working remotely after the end of the COVID-19 emergency.

Low back pain was assessed by the Brief Pain Inventory (BPI) [17]. The pain intensity section of the BPI is composed by four items that are scored from 0 (no pain) to 10 (worst pain), while the functional interference section is composed by seven items that are scored from 1 (no interference with activities of daily living, ADLs) to 10 (total interference). The severity index is calculated on the basis of the mean of the four pain intensity items, and interference index is calculated from the mean of the seven pain interference items. 

Workers’ beliefs about how physical activity and work affect LBP and neck pain were rated using the Fear Avoidance Belief Questionnaire (FABQ), which consists of 16 items investigating how physical activity and work affect employees’ pain [18]. The FABQ Physical Activity (FABQ-PA) evaluates atti tudes and beliefs related to general physical activities (five items, range 0–30), and the FABQ Work (FABQ-W) assesses attitudes and beliefs related to occupational activities (eleven items, range 0–66). Each item is scored from 0 (“do not agree at all”) to 6 (“completely agree”). The overall score is calculated by adding FABQ-PA and FABQ-W scores (range 0–96). 

Job satisfaction was assessed by the Utrecht Work Engagement Scale (UWES) [19]. This tool includes 17 items divided into three dimensions (i.e., vigor, dedication, and absorption). Items were measured on a 7-point rating scale, from 0 (never) to 6 (always).

**Figure 1:**
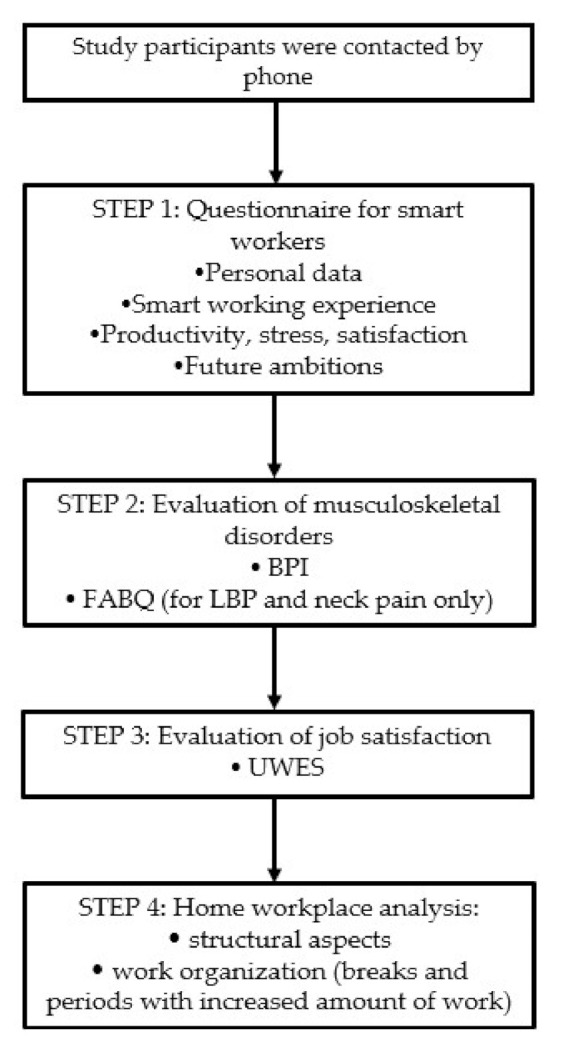
Flow chart of the evaluation protocol.

Participants were asked about structural aspects of their workplace at home: chair (adjustable seat height, back height, back inclination), table (type, height), type of computer (desktop/laptop), monitor (adjustable in inclination, height, rotation), eye distance from the monitor, presence of external keyboard and its distance from the edge of the table, presence of a footstool. Presence of breaks and periods with increasing amount of work were also investigated. For the general health risk assessment, we referred to current regulations and the Italian Organization for Standardization (UNI) standards (UNI EN 1335-1—“Office work chairs—Dimensions—determination of dimensions”; UNI EN 527-1 “Office furniture—Work tables and desks—dimensions”; UNI 10380-A1 “Indoor lighting with artificial light”) (Figure 1). 

### 2.3. Statistical Analysis

Descriptive statistical analy sis was performed using the SPSS v. 25.0 software (SPSS Inc.; Chicago, IL, USA). Continuous variables are expressed as means ± standard deviations, while categorical ones are reported as absolute values and percentages, whereas the ordinal data are represented as medians. We performed the Shapiro–Wilk normality test for all the continuous data. If data followed a normal distribution, the Student’s *t* test was used to compare data across groups; if not, the two-sample Wilcoxon rank-sum (Mann–Whitney) test was used when appropriate. Statistical tests were carried out on a two-sided significance level of 0.05.

## 3. Results

A total of 51 home workers were included. The mean age was 46.67 ± 11.26 years, and the percentage of women was 56.9%. Most of the participants had three or more cohabitants (56.9%), but only 29.4% had children to look after. Fifty-five percent of workers had a second level degree. The main population characteristics are reported in Table 1. 

In 53% of cases, no differences were recorded between home and office working in terms of tasks, schedule, and salary. Thirty-nine percent of the subjects self-perceived to be less productive but less stressed, while 51% were equally satisfied. Among mobile working advantages, the most appreciated was saved travel time (82.4%) and the least appreciated was greater autonomy (9.8%). Impaired interaction with colleagues (41.2%) and distractions in the domestic environment (40.6%) were judged to be the worst disadvantages.

Thirty-nine percent of employees stated that they would like to continue working at home only occasionally. Characteristics and quality of remote work are showed in Table 2.

Concerning health problems, 70.5% of participants reported MSK pain, most frequently at the low back (41.2%) or neck (23.5%), and 23.5% in multiple sites (Table 3). 

Pain severity and pain interference during everyday activities have been found slightly higher for neck pain compared to LBP (Table 4). 

In the FABQ subscales, the mean score was higher in the work component than in the physical activity component for subjects affected by LBP or neck pain. Moreover, workers with neck pain reported a higher mean score on the FABQ work component than those with LBP (Table 5).

Worsening of previous neck pain was reported by 50% of participants, while in 8.3% an improvement of neck pain occurred.

In 47.6% of subjects, there was no exacerbation of LBP since they work remotely, whereas 38.1% reported an increase of LBP severity, and only 14.3% showed pain improvement (Table 6).

Home workers without pain reported a significantly higher job satisfaction assessed by UWES than those with pain (*p* = 0.009) (Table 7).

Regarding structural aspects of the home workplace (Table 8 and Table 9), most of the participants reported using a conventional four-leg kitchen chair (56.9%), and that the seat was not adjustable in height (54.9%). In most cases, the back was concave (54.9%), not adjustable in height (70.6%) or inclination (68.6%). Although most workers used a worktable with height 72 ± 1.5 cm (home table), 39.2% used the kitchen table (height over 73.5 cm). In 86.3% of cases, the table had a single top not adjustable in height.

The number of home workers who used a desktop computer was higher (58.8%), with monitor adjustable in height only for 29.4%. In two-thirds of cases, eye distance from the monitor was 50–70 cm. External keyboard was used by 62.7% of individuals, and in almost all workers (92.2%) there was enough space for the upper limbs as the keyboard was positioned 15 cm away from the table edge. Nobody used a footstool. Forty-one percent used a laptop. Finally, all participants reported taking self-managed breaks. Periods with increasing amount of work were reported by 51% of home workers.

## 4. Discussion

To the best of our knowledge, this is the first study investigating how home workers set up their home workplace and the impact of existing equipment on MSK health. Moreover, no previous study had ever measured mobile working-related job satisfaction on a specific scale.

We characterized a population of mobile workers in terms of work-related outcomes, such as perceived productivity and job satisfaction, and onset or changes of previous MSK disorders, particularly LBP and neck pain.

Over 80% of workers reported no difference in tasks, although 29.4% had a different schedule, with 51% of the population declaring, surprisingly, less than 36 working hours per week, according to regular hours of employment for office workers in Italy.

### 4.1. Productivity

In our population, working at home resulted in relevant productivity changes (a decrease in 39.2% and an increase in 29.4% of participants). These data are in contradiction with results publicized by FlexJobs’ 7th annual survey, where about 65% of workers assessed their productivity as higher at home than in a traditional office [20]. The reduction of productivity in our study could be explained by the presence of distractions in the domestic environment and impaired interaction with colleagues, whereas in participants reporting increasing productivity, a main role may be played by reduced stress and/or commuting time.

### 4.2. Job Satisfaction

In our population, about half of the participants did not report any variation in job satisfaction between remote and office work. This finding might likely be due to unchanged job type and amount during the home working period. Our data are consistent with that of other studies [21,22] demonstrating a negative correlation between job satisfaction and the increased amount of home working.

### 4.3. Mental Health

Forced social isolation coupled with a marked reduction in physical activity could negatively impact both physical and mental health [23,24,25]. Therefore, remote working seems to also be associated with an increased risk of mental and physical health issues.

Regarding occupational stress, no significant change occurred in mobile workers, considering that 39.2% of participants declared a reduced stress level since they work remotely, 27.5% reported an unchanged level, and one-third of subjects experienced increased stress. On the contrary, in the research conducted by the International Labour Organization and Eurofound, about 41% of home workers declared that they felt stressed compared with 25% of their colleagues who work in the office [1]. The stress reduction reported in our study could be due to saved travel time to go to the office, higher time flexibility, and better family life.

### 4.4. Physical Health

Concerning physical health issues related to remote working, increased sedentariness and poor posture due to the use of non-ergonomic equipment in our population seemed to promote the onset of MSK disorders, particularly LBP and neck pain. This finding is not surprising, considering that spine pain is one of the most frequent health problems in the working-age population worldwide. According to a recent study, the prevalence and incidence of LBP ranged from 1.4% to 20% and from 0.024% to 7%, respectively, in workers [26]. The overall mean prevalence of neck pain in the general population is 3.6% [27], with a higher incidence in office and computer workers [28]. Italian estimated lifetime prevalence is 9% for LBP and 5% for neck pain [29].

Literature offers controversial evidence about the relationship between LBP and sedentary jobs. It has been argued that the risk of LBP seems to increase when office workers stay seated for more than 7 h per day. However, no significant association between sitting itself and the risk of LBP has been demonstrated [30]. This finding could be explained by the multifactorial nature of LBP [31]. The incidence of this condition is significantly associated with anthropometric, ergonomic, and psychosocial factors, in particular age, gender, body mass index, body distance from computer screen, adjustable back support, body position while sitting, job satisfaction, and repetitive work [32]. 

As stated by Burdorf et al., a sustained sedentary job in a forced non-neutral trunk posture is a risk factor for LBP [33]. Due to low-grade activation of lumbar muscles while sitting, the load is conducted by passive structures such as ligaments and intervertebral discs. Because of the viscoelasticity of these structures and deactivation of lumbar muscles, the lumbar spine may be predisposed to deconditioning and LBP [34]. 

Similarly, while an association between the increased use of computers and work-related neck pain has been observed, it is unclear whether this is a causal relationship, considering the complex etiology of neck pain that comprises physical, psychological, and environmental factors [35].

Office workers with neck pain usually show limited range of motion of the cervical spine and enhanced activity of the cervical flexors and cervical extensors muscles [36], which might prolong neck pain.

A comfortable workplace may help in preventing MSK disorders. Some experts recommend [37] that the worktable and chair must be adjustable in height so that the feet are supported to be always well placed on the ground. In the absence of a height-adjustable chair, the use of a footrest is recommended. Moreover, the monitor must be at the appropriate eye level so as not to force a persistent head tilt. In our study, we found that most of the participants used a common kitchen chair, not adjustable in height, and nobody used a footrest during working hours. Therefore, these factors may contribute to LBP, although no relevant changes in the onset and/or worsening of this condition were reported in our population. On the other side, both frequency (23.5%) and worsening (50%) of neck pain were stepped up in workers who used laptops without any height-adjustable support. 

However, pain intensity and interference with ADL (BPI scores) seemed to be negligible in home workers with LBP or neck pain. Furthermore, the work component of FABQ in people reporting low back and neck pain was mild. These data testify that remote working seems to not significantly affect spine pain, probably because subjects were practicing this job type since about 3 months, a too brief period to produce the putative adverse effects of prolonged use of non-ergonomic equipment. 

According to a national survey [38], LBP and neck pain got worse during the lockdown, and 21% of individuals attributed this worsening to home working. Our results are consistent with these data only for neck pain, while the participants have not declared a worsening of LBP during home working period. 

Furthermore, participants with pain were less satisfied with working at home. However, 62.7% of participants expressed the wish to continue remote working in the future, at least occasionally. 

### 4.5. Limitations

The first limitation of our study is the small sample size, which can lead to unpersuasive findings. Second, our results refer to Italian workers from the Campania Region, so they may be not generalizable across different regions or countries. Third, the cross-sectional design is not able to establish a cause–effect relationship because exposure and outcome are simultaneously assessed. Finally, productivity, satisfaction, and MSK issues in workers often have a variety of simultaneous influences that need to be accounted for.

## 5. Implications for Practice

In the face of increasing use of home working, we did not find an adequate home environment, with a higher risk for health issues, particularly affecting the spine. Our study suggests solutions for adapting the home environment, such as adjustable seating and worktable, which in our opinion can significantly reduce risks for health. This would lead to better productivity, lower costs, and enhanced job satisfaction. Our results show reduced remote workers’ perception about productivity. Effective organization of the working day at home may improve job performance. It could be nice to make a list of daily goals, to create a space specifically reserved for work, and to reduce sources of distraction (i.e., by family members). In our perspective, management strategies should be provided to enhance productivity, particularly by adapting the home environment to allow comfortable working posture (height-adjustable chairs, tables, and monitors). These adjustments might lead to improving overall health and job performance.

## 6. Conclusions

The COVID-19 outbreak and social distancing have radically changed the work organization. Current research investigated the impact of mobile working on work-related outcomes, mental health, and MSK issues. In this survey, we have investigated for the first time how remote workers set up their home workplace and the impact of existing equipment on LBP and neck pain.

In our study, home workers perceived themselves to be less productive, less stressed, and equally satisfied compared to their office working period. Remote workers appreciated particularly saved travel time to go to work and were not pleased to be isolated from colleagues. 

The use of non-ergonomic equipment (conventional four-leg kitchen chair not adjustable in height, not height-adjustable monitor, the absence of a footstool) may increase MSK disorders.

Most participants complained of worsening of neck pain, but no exacerbation of LBP was reported, probably due to the short duration of the study. Moreover, our results suggest that MSK disorders related to remote working might reduce job satisfaction.

Data provided by this survey would be useful to improve the home working environment and time organization in order to promote the mental and physical health of remote workers.

Further studies with a greater sample size are needed to examine, in a longer time span, the risks for MSK well-being and the health-related burden of home working. 

## Figures and Tables

**Table 1:** Study population characteristics.

Study Participants	Total (*N* = 51)
Age (years)	46.67 ± 11.26
Weight (kg)	72.69 ± 13.57
Height (cm)	168.82 ± 8.43
BMI (kg/m^2^)	25.41 ± 4.29
Gender	
Male	22 (43.1%)
Female	29 (56.9 %)
Cohabitants	
≥3	29 (56.9%)
<3	22 (43.1%)
Minor children	
No	36 (70.6%)
Yes	15 (29.4%)
Education	
Primary School	0 (0%)
Secondary School	0 (0%)
High School	17 (33.3%)
First Level Degree	4 (7.8%)
Second Level Degree	28 (55%)
University Master	1 (1.9%)
PhD	1 (1.9%)

Note: Values are expressed as means ± standard deviations for continuous data and counts (percentages) for categorical data. BMI, body mass index.

**Table 2:** Characteristics and quality of remote work.

Home Working Feature	Total (*N* = 51)
Type of remote work	
Same as office work	27 (53%)
Different tasks	8 (15.7%)
Different schedule	15 (29.4%)
Different salary	1 (1.9%)
Working hours per week	
<36	26 (51%)
≥36	25 (49%)
Productivity	
Lower	20 (39.2%)
Equal	16 (31.4%)
Higher	15 (29.4%)
Stress	
Lower	20 (39.2%)
Equal	14 (27.5%)
Higher	17 (33.3%)
Satisfaction	
Lower	18 (35.3%)
Equal	26 (51%)
Higher	7 (13.7%)
Advantages *	
Saved travel time	42 (82.4%)
Time flexibility	12 (23.5%)
Greater autonomy	5 (9.8%)
Time spent with family	13 (25.4%)
Enhanced attention	6 (11.8%)
Disadvantages *	
Distractions in the domestic environment	20 (40.6%)
Planning difficulties	5 (9.8%)
Impaired interaction with colleagues	21 (41.2%)
Technical failures	12 (23.5%)
Desire to continue home working	
Yes, as much as possible	12 (23.5%)
Yes, occasionally	20 (39.2%)
No, for difficult job management	0 (0%)
No, for increased costs	0 (0%)
No, for the lack of interaction with colleagues	16 (31.4%)
No, for the increase in distraction factors	3 (5.9%)
No, for increased work amount	0 (0%)

Note: Values are expressed as counts (percentages). * For these items, more than one answer was possible.

**Table 3:** Sites of home working-related pain *.

Site	Total (*N* = 51)
Low back	21 (41.2%)
Neck	12 (23.5%)
Shoulder	4 (7.8%)
Hip	4 (7.8%)
Knee	4 (7.8%)
Thigh	3 (5.9%)
Elbow	2 (3.9%)

Values are expressed as counts (percentages). * 23.5% of participants referred multiple sites of pain.

**Table 4:** Home working-related pain measured with the Brief Pain Inventory (BPI).

Site	BPI Severity Index	BPI Interference Index
Low back pain	1.52 ± 1.63	2.28 ± 1.33
Neck pain	1.97 ± 1.70	2.75 ± 1.78
Shoulder pain	1.40 ± 1.06	1.97 ± 1.26
Hip pain	0.80 ± 1.04	1.95 ± 1.44
Knee pain	1.43 ± 0.98	2.06 ± 1.38
Thigh pain	1.20 ± 0.50	0.95 ± 0.08
Elbow pain	3.05 ± 0.63	2.95 ± 1.20

Values are expressed as means (SD). BPI, Brief Pain Inventory.

**Table 5:** Low back and neck pain measured with the Fear Avoidance Beliefs Questionnaire (FABQ).

	FABQ-PA	FABQ-W	FABQ-TOT
Low back pain	10.10 ± 5.96	11.52 ± 11.04	21.62 ± 13.67
Neck pain	10.67 ± 6.37	14.08 ± 10.46	24.75 ± 14.56

Values are expressed as means (SD). FABQ-PA, Fear Avoidance Beliefs Questionnaire—Physical Activity; FABQ-W, Fear Avoidance Beliefs Questionnaire—Work; FABQ-TOT, Fear Avoidance Beliefs Questionnaire—Total.

**Table 6:** Impact of home working on musculoskeletal disorders (Total = 51).

	Low Back Pain*N* = 21	Neck Pain*N* = 12	Shoulder Pain*N* = 4	Hip Pain*N* = 4	Knee Pain *N* = 4	Thigh Pain *N* = 3	Elbow Pain *N* = 2
Improved	3 (14.3%)	1 (8.3%)	0(0%)	0 (0%)	0 (0%)	0 (0%)	0 (0%)
Worsened	8 (38.1%)	6 (50%)	2 (50%)	1 (25%)	0 (0%)	0 (0%)	2 (100%)
Equal	10 (47.6%)	5 (41.7%)	2 (50%)	3 (75%)	4 (100%)	3 (100%)	0 (0%)

Values are expressed as counts (percentages).

**Table 7:** Job satisfaction assessment.

	UWES-17	*p*-Value
Home workers with pain (*N* = 36)	74.86 ± 14.42	0.009 *
Home workers without pain (*N* = 15)	87.70 ± 9.10	
Total home workers (*N* = 51)	78.17 ± 16.29	

Values are expressed as means (SD). UWES, Utrecht Work Engagement Scale. * Two-sample Wilcoxon rank-sum (Mann–Whitney) test.

**Table 8:** Home working equipment characteristics: work chair and table.

Equipment	Total (*N* = 51)
Chair	
Adjustable seat height	
Yes	23 (45.1%)
No	28 (54.9%)
Support	
4 legs	29 (56.9%)
5 wheels	22 (43.1%)
Back	
Flat	23 (45.1%)
Concave	28 (54.9%)
Adjustable back height	
Yes	15 (29.4%)
No	36 (70.6%)
Adjustable back inclinationYes	16 (31.4%)
No	35 (68.6%)
Table	
Type of table	
Single top not adjustable in height	44 (86.3%)
Single or double top adjustable in height	6 (11.8%)
Two-height top with lowered keyboard holder	1 (1.9%)
Table height	
Over 73.5 cm	20 (39.2%)
72 ± 1.5 cm	24 (47%)
Under 70.5 cm	7 (13.8%)

Values are expressed as counts (percentages).

**Table 9:** Home working equipment characteristics: computer and keyboard.

Equipment	Total(*N* = 51)
Desktop/Laptop	
Desktop	30 (58.8%)
Laptop	21(41.2%)
Monitor	
Not adjustable	6(11.8%)
Adjustable in inclination	30 (58.8%)
Adjustable in inclination, height, rotation	15 (29.4%)
Eye distance from the monitor	
<50 cm	9 (17.7%)
50–70 cm	34 (66.6%)
>70 cm	8 (15.7%)
Keyboard distance from the edge	
<15 cm	4 (7.8%)
>15 cm	47 (92.2%)
External keyboard	
No	19 (37.3%)
Yes	32 (62.7%)

Values are expressed as counts (percentages).
